# Preparation of Ni_3_Fe_2_@NC/CC Integrated Electrode and Its Application in Zinc-Air Battery

**DOI:** 10.3389/fchem.2020.575288

**Published:** 2020-11-09

**Authors:** Hui Hu, Xiaofei Ling, Chaogui Tan, Jianguo Lin, Xiaopeng Han, Wenbin Hu

**Affiliations:** ^1^School of Materials Science and Engineering, Xiangtan University, Xiangtan, China; ^2^Tianjin Key Laboratory of Composite and Functional Materials, School of Materials Science and Engineering, Tianjin University, Tianjin, China

**Keywords:** zinc-air battery, integrated electrode, NiFe nanoparticle, nitrogen-doped carbon, OER/ORR

## Abstract

Reasonable design and development of a low-cost and high-efficiency bifunctional electrocatalyst for oxygen evolution reaction (OER) and oxygen reduction reaction (ORR) is essential for promoting the development of Zinc-air battery technology. Herein, we obtained an integrated catalytic electrode, NiFe nanoparticles supported on nitrogen-doped carbon (NC) directly grown on the carbon cloth (designated as Ni_3_Fe_2_@NC/CC), by pyrolysis of bimetallic NiFe metal-organic framework (MOF) precursor. There is a synergistic effect between nickel and iron component, which enhances the bifunctional catalytic activity. In addition, the underlying carbon cloth is conducive to the efficient electron transfer and also benefits the uniform loading of catalytically active materials. Thus, the integrated electrode shows good OER/ORR dual-functional catalytic performance, and the OER overpotential is much lower than that of the traditional drop-coating electrode and precious metal catalyst (IrO_2_). Moreover, the Ni_3_Fe_2_@NC/CC integrated electrode used in zinc-air batteries shows good flexibility and cycle stability. Our findings provide a new avenue for the development of efficient and stable bifunctional oxygen electrocatalysts.

## Introduction

The crisis from environmental and energy issues have driven the research and development of new energy technologies on a global scale (Poizot and Dolhem, 2011; Larcher and Tarascon, 2015; Chu et al., 2016; Stamenkovic et al., 2016; Li and Wang, 2019; Chen et al., 2020). Among new energy technologies, sustainable energy conversion and storage technologies such as fuel cells, electrolyzed water devices, and metal-air batteries are developing rapidly (Anantharaj et al., 2016; Lee et al., 2016a; Zhang et al., 2016; Li and Lu, 2017; Yu et al., 2019; Wang et al., 2020). The metal-air battery has received increasing attention attribute to the advantages of low cost, high specific energy density, remarkable long-term stability, and environmental benignity (Fu et al., 2017b; Pan et al., 2018; Xiong and Ivey, 2018). Moreover, the theoretical energy density of metal-air battery is 5~10 times than that of lithium-ion batteries (Gu et al., 2017). Metal-air batteries include a metal anode (metal=lithium, zinc, magnesium, aluminum, etc.) and an air cathode. On the one hand, the metal-air battery is suitable for practical applications due to its abundant resources, low price, and environmental friendliness (Chen et al., 2018; Wang et al., 2019). On the other hand, the battery performance can be further improved by optimizing the electrode reaction kinetic of the air cathode (Cheng and Chen, 2012; Lee et al., 2016a). During the charge-discharge process, the electrode reactions on the air cathode are oxygen evolution reaction (OER) and oxygen reduction reaction (ORR) (Jiang et al., 2016; Han et al., 2017; Suen et al., 2017). Rational design and synthesis of catalytic materials to promote the process of OER/ORR have attracted the interest of many researchers.

So far, precious metals and their alloys are still considered to be state-of-the-art electrocatalysts for OER/ORR, such as Pt-based material catalysts with high ORR activity, Ir-based or Ru-based material catalysts with high OER activity (Lee et al., 2012; Liu et al., 2019a). However, the prohibitive cost, low reserves, and poor stability severely restrict the large-scale production and commercial application. Since transition metals (such as iron, cobalt, and nickel) based catalysts have the characteristics of low cost, earth-abundance, and good chemical stability, they are recognized as ideal materials to replace noble metals (Han et al., 2019a; Shi et al., 2019). In recent years, there has been a lot of research results on transition metal catalysts (Aijaz et al., 2016; Han et al., 2016). Transition metal, transition metal alloys and their derivatives (such as oxides, hydroxides and carbides) are developed as low-cost oxygen electrocatalysts, which have become a kind of substitute for precious metals and have attracted widespread attention (Gong et al., 2013; Cai et al., 2016; Fu et al., 2017a; Liu et al., 2019b; Xie et al., 2019). However, a large number of experimental studies have shown that it is difficult for a single metal catalyst to meet the bifunctional catalytic requirements (Liang et al., 2013; Park et al., 2017; Li et al., 2018). Therefore, the development of OER/ORR bifunctional catalysts based on earth-abundant elements with satisfied electrochemical activity and excellent stability still remains a great challenge.

The synergistic effect between different metals in bimetallic materials can effectively change the electronic structure of materials and reduce the free energy of reaction, leading to the effective bifunctional capability (Su et al., 2017). Besides, rational design of catalytic electrode structure is an important approach to improve the electrochemical performance. The traditional electrode preparation process is to disperse the powdered catalyst in a solvent and prepare an electrode slurry, which is then coated on carbon cloth or carbon paper to prepare the final air cathode. The preparation method not only makes the catalyst loaded on the support non-uniform, but also causes the weak interaction between the functional phase and the support, resulting in poor electrode stability. Moreover, the binder added in the preparation of electrode slurry will cover the catalytic active site and increase the interfacial resistance, unfavorable for the activity enhancement.

In this work, a novel NiFe nanoparticles supported on nitrogen-doped carbon (NC) hybrid material, directly grown on the carbon cloth (designated as Ni_3_Fe_2_@NC/CC), was proposed and synthesized as an integrated electrode for promoting OER/ORR electrocatalysis and zinc-air batteries. The designed synthetic strategy includes a chemical precipitation method and then a one-step pyrolysis procedure. Compared with the traditional electrode in which Ni_3_Fe_2_@NC is prepared as an electrode slurry drop-coated on carbon cloth, the Ni_3_Fe_2_@NC/CC integrated electrode exhibits greatly improved catalytic activity. When the anodic current density reaches 10 mA cm^−2^, the OER overpotential is 238 mV, which is lower than the Ni_3_Fe_2_@NC drop coating electrode (340 mV) and the precious metal oxide IrO_2_ (400 mV). The Ni_3_Fe_2_@NC/CC also exhibits remarkable long-term catalytic durability. It can be directly used as the positive electrode for practical aqueous and flexible semi-solid zinc-air batteries, which deliver large discharging capacity, more than 80 discharging-charging cycles and good flexibility.

## Experimental Section

### Materials Synthesis

In a typical synthetic procedure, potassium hexamethylene ferrite (1.33 g) was put in a 200 mL beaker, and then added in 100 mL deionized water and pre-treated carbon cloths to form settled solution A. In the meantime, nickel nitrate hexahydrate (1.74 g) and trisodium citrate (2.36 g) were dissolved into 200 mL water to form clear solution B. Solution A and solution B were mixed under strong magnetic stirring to form a homogeneous solution. After 24 h standing and ripening, NiFe-MOFs coated carbon cloths were taken out to dry at room temperature. At the same time, the solution was centrifuged to obtain the solid, washed with water and ethanol for three times, and then dried at room temperature to achieve the NiFe-MOFs powder. The NiFe-MOFs carbon cloths and powders were transferred into the porcelain boat. And then, the boat was placed in a tube furnace, heated to 500°C with a slow heating rate of 2°C min^−1^ under Ar atmosphere and kept for 1 h. After the temperature was naturally down to room temperature, the obtained integrated electrode and powder sample were collected and signed as Ni_3_Fe_2_@NC/CC and Ni_3_Fe_2_@NC, respectively.

### Materials Characterization

The phase and crystal structure were characterized by an X-ray diffraction analyzer (XRD, Bruker D8 Advanced, CuKα radiation). Scanning electron microscope (SEM, s4800 Hitachi, 30 kV) with energy dispersive spectrometer (EDS), transmission electron microscope (TEM, JEOL JEM-2100F, 200 kV) and high-angle annular dark-field scanning transmission electron microscope (HAADF-STEM, JEM-ARM200F, 200 kV) were used to observe the microstructure and nanostructure of samples. The chemical valence and surface composition were detected by Thermo Scientific X-ray photoelectron spectroscopy (XPS, Escalab 250Xi).

### Electrocatalytic Measurements

The electrochemical performance was tested at the IviumStat workstation. The electrochemical test is carried out in a three-electrode system, including a working electrode, a reference electrode, and a counter electrode. The integrated Ni_3_Fe_2_@NC/CC electrode prepared in this work can be directly used as the working electrode. The saturated calomel electrode is the reference electrode, and the carbon rod or platinum is the counter electrode. The obtained Ni_3_Fe_2_@NC powder samples were made into working electrodes according to traditional electrode preparation methods for comparison. Electrode slurry was prepared by suspending 10 mg Ni_3_Fe_2_@NC in a mixed solvent of deionized water 0.68 mL, isopropanol 0.23 mL, and Nafion 0.09 mL (v/v/v = 15/5/2). For noble metal catalysts, 8 mg IrO_2_ was mixed with 2 mg carbon powder. The OER working electrode was prepared by the drop-casting method, and the slurry was deposited on 1.0 × 1.0 cm^2^ carbon cloth (loading mass is about 1.0 mg cm^−2^) and dried at 60°C. The OER performance was tested in 1M KOH saturated with high purity N_2_. IrO_2_ electrode is also prepared by the same process. The ink of the catalyst was deposited on the glass carbon of the rotating disk electrode (RDE) with a diameter of 5 mm to prepare an ORR working electrode. Then, 10 μL of the resulting ink was coated on the electrode and dried in the air environment. The ORR performance of the catalyst was measured in 0.1 M KOH solution saturated with oxygen. First, several cyclic voltammetry (CV) curves were performing until the signal was stable. Linear sweep voltammetry of ORR and OER was performed at a scan rate of 10 mV s^−1^. The electrochemical impedance spectroscopy is performed in the frequency range of 100 kHz to 100 mHz. The electric double-layer capacitance (C_dl_) was measured by CV. For data analysis, convert the potential to a reversible hydrogen electrode (RHE) according to the following formula: E (vs. RHE) = E (vs. SCE) + 0.059 × pH + 0.241 V. The OER polarization measurements were iR-corrected. The Koutecky-Levich (K-L) curve is obtained by the following formula:

$$\begin{array}{l} \frac{1}{I}=\frac{1}{I_{k}}+\frac{1}{I_{d}}=\frac{1}{nFAkC^{0}}-\frac{1}{0.62nFAD_{O_{2}}^{2/3}v^{-1/6}C^{0}\omega^{1/2}} \end{array}$$

Among them, I, I_k_ and I_d_ represent measurement, kinetics and limit diffusion current density, n is the number of electrons transferred by oxygen reduction, F is the Faraday constant, A (cm^2^) is the geometric area of the electrode, k is the rate constant of the reaction, and C^0^ is the oxygen constant in a saturated 0.1 M KOH solution, D is the oxygen diffusion coefficient, ν is the solvent dynamic viscosity, and ω is the speed in rad s^−1^.

### Assembly of Aqueous Zn-Air Batteries

The aqueous zinc-air battery was constructed by the anode, the cathode, and the electrolyte. The anode of the battery was a zinc plate, the electrolyte was 6.0 M KOH and 0.2 M ZnCl_2_ solution, and the air cathode was a catalyst coated on a 1 × 1 cm^2^ carbon cloth. The integrated electrode obtained in this work was directly used as an air cathode. Before testing, high-purity oxygen must be allowed to enter the electrolyte for about 20 min to reach the oxygen saturation state. During the test, oxygen should be continuously introduced at a small flow rate to ensure oxygen saturation of the electrolyte. The open-circuit voltage and reversible cycle of the zinc-air battery were tested by the LAND-CT2001A testing device.

### Assembly of Flexible Semi-Solid Zn-Air Batteries

The flexible semi-solid zinc-air battery was composed of a zinc anode, a Polyvinyl alcohol (PVA) KOH solid electrolyte, and Ni_3_Fe_2_@NC/CC air cathode. The preparation method of the PVA-KOH electrolyte was shown below. Dissolve 3 g of PVA powder in 25 mL of deionized water and stir thoroughly at 90 °C to form a clear solution. Then add 6 mL 9 M KOH solution to mix, continue to stir at 90°C for 4 h, then put in the refrigerator to freeze for at least 3 h.

## Results and Discussion

**Figure 1:**
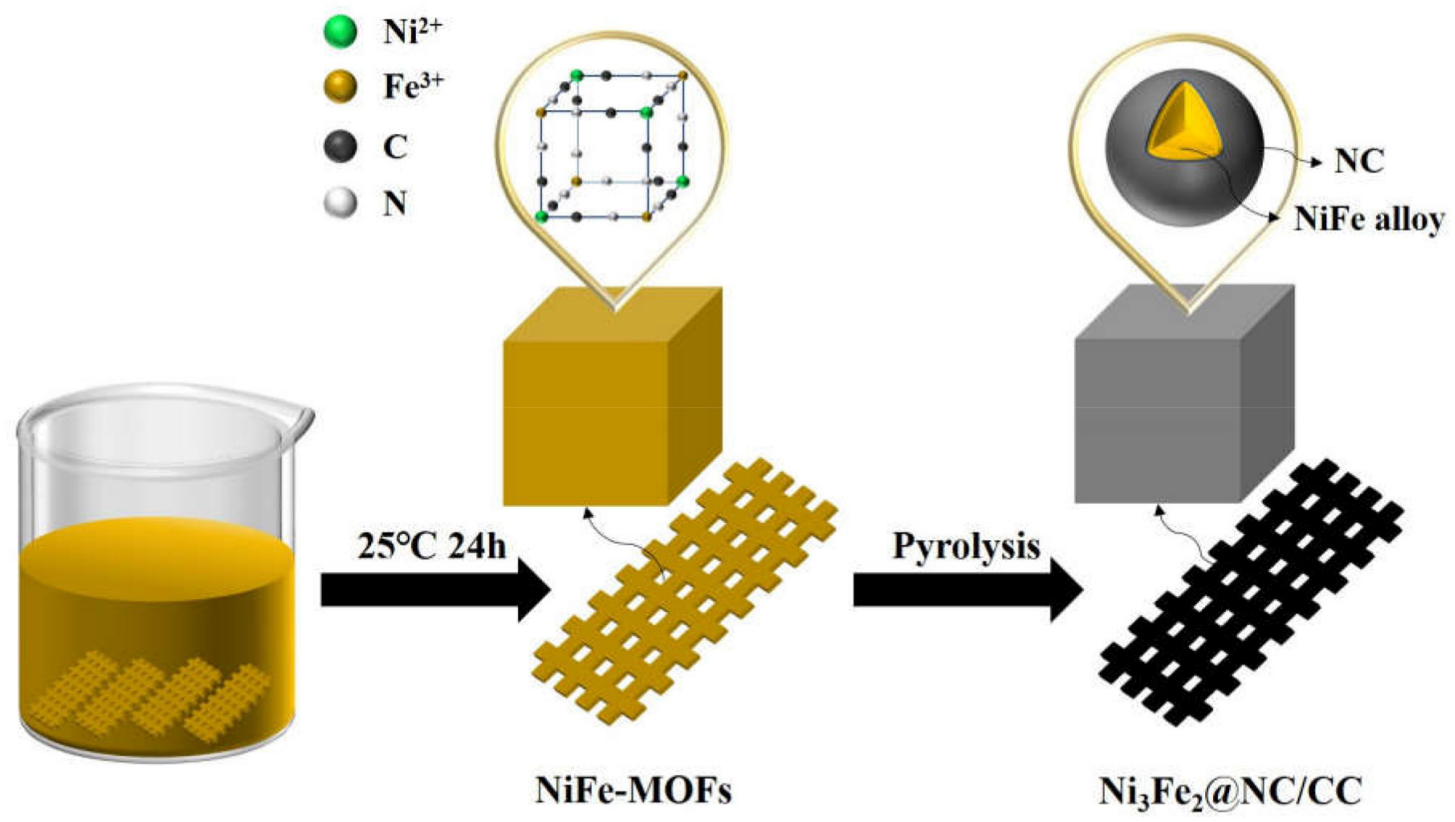
Illustration of the preparation procedures of Ni_3_Fe_2_@NC/CC.

**Figure 2:**
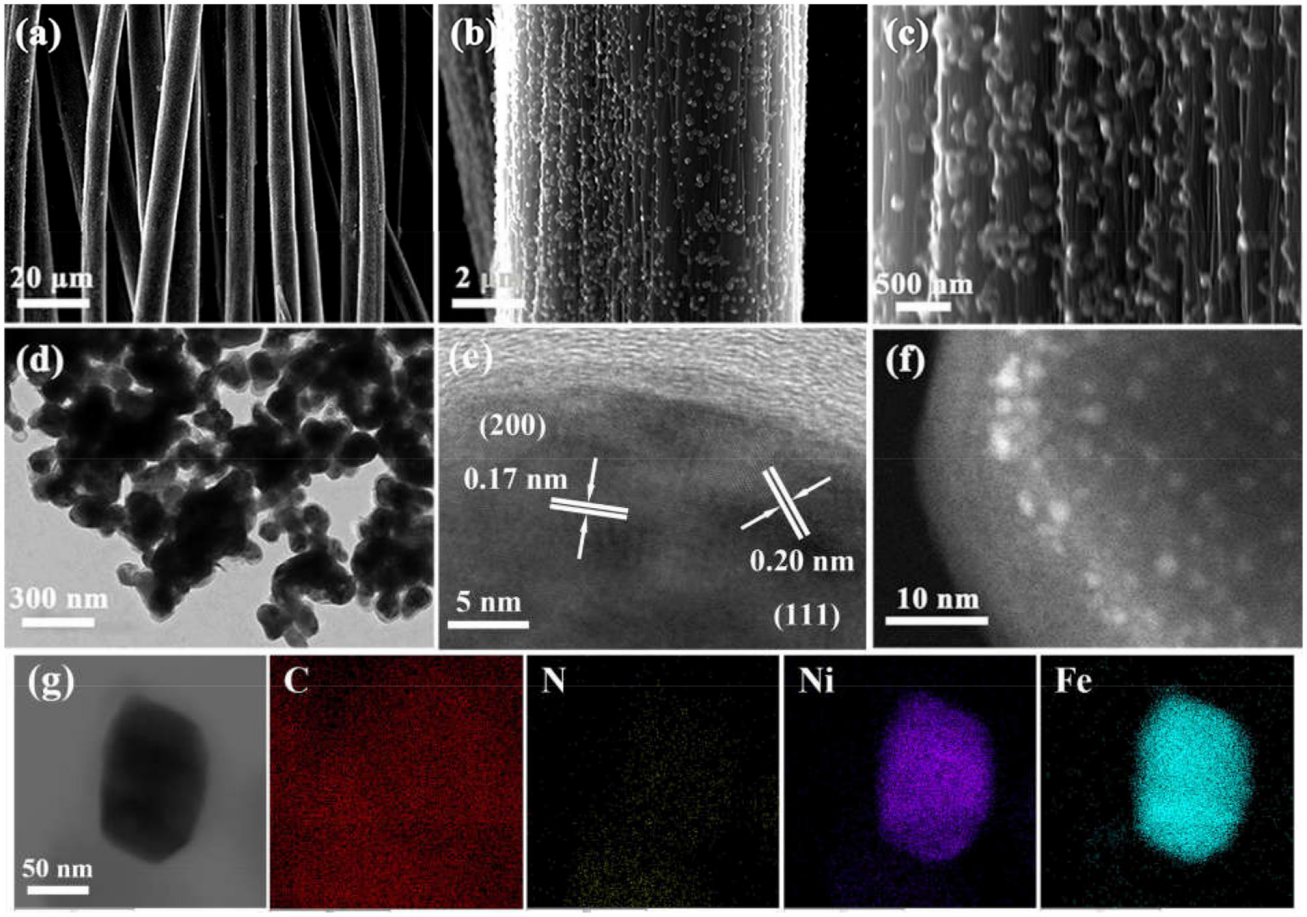
**(a–c)** SEM images from low to high magnification of Ni_3_Fe_2_@NC/CC. **(d)** TEM, **(e)** HRTEM, **(f)** HAADH-STEM, and **(g)** elemental mapping images of Ni_3_Fe_2_@NC.

Figure 1 schematically elucidates the preparation process of the Ni_3_Fe_2_@NC/CC. In brief, nickel (II) nitrate hexahydrate, sodium citrate, and potassium ferricyanide are co-precipitated in deionized water to form NiFe-MOFs precursor. Carbon cloths are added during the precipitation formation. Ni_3_Fe_2_@NC and Ni_3_Fe_2_@NC/CC are finally achieved by calcining the precursor and the carbon cloth under the argon atmosphere at 773 K, respectively. The NiFe-MOFs nanoparticles with particle size about 100 nm uniformly loaded on the carbon fiber (Supplementary Figures 1A–C, Supporting Information). The XRD pattern of the precursor that had been sonicated from the carbon cloth was consistent with the theoretically fitted Ni_3_[Fe(CN)_6_]_2_·H_2_O, (Zhang et al., 2017), which means the successful formation of the NiFe-MOFs precursor (Supplementary Figure 2). After calcination at 773 K under an argon atmosphere, the organic precursors were carbonized into NC and the nanoparticles were anchored on the NC matrix. XRD analysis of the resultant composites elucidated that the dominant phase was bimetal NiFe with three obvious diffraction peaks at 44, 51, and 75°, which can be assigned, respectively, to the (111), (200), and (220) planes of NiFe alloy (Supplementary Figure 3A). Energy dispersive spectrum (EDS) showed that the atomic ratio of Ni and Fe in the calcined sample was 3:2 (Supplementary Figure 3B and Supplementary Table 1). Comprehensive analysis of XRD and EDS data demonstrates the successful synthesis of nickel-iron nanoparticles on the N-doped carbon nanocube structure. Furthermore, as shown in Figures 2a–c, Ni_3_Fe_2_@NC nanoparticles evenly loaded on the carbon cloth with the average particle size of ~100 nm. For further structural analysis of nanoparticles on carbon cloth, put the calcined integrated electrode in absolute ethanol and sonicate, and the dissolving sample was taken to perform TEM test. TEM further confirms the nanoparticles with the size of about 100 nm (Figure 2d). The high-resolution TEM (HRTEM) image proved distinct domains of NiFe alloy with the interplanar spacing of 0.20 and 0.17 nm, corresponding to the (111) and (200) facets of NiFe alloy, respectively (Figure 2e). To further characterize the NiFe alloy, an aberration-corrected high-angle annular dark-field scanning transmission electron microscope (HAADF-STEM) test was performed on it. The HAADF-STEM image showed NiFe alloy with a heavier element mass on an NC substrate exists in the form of bright spots, and the particle size of the NiFe alloys is about 2 ~ 3 nm (Figure 2f). Also, elemental mapping of Ni_3_Fe_2_@NC revealed the homogeneous dispersion of C, N, Ni, and Fe species throughout the entire nanoarchitecture (Figure 2g), suggesting the uniform loading of NiFe nanoparticles on N-doped carbon structure.

**Figure 3:**
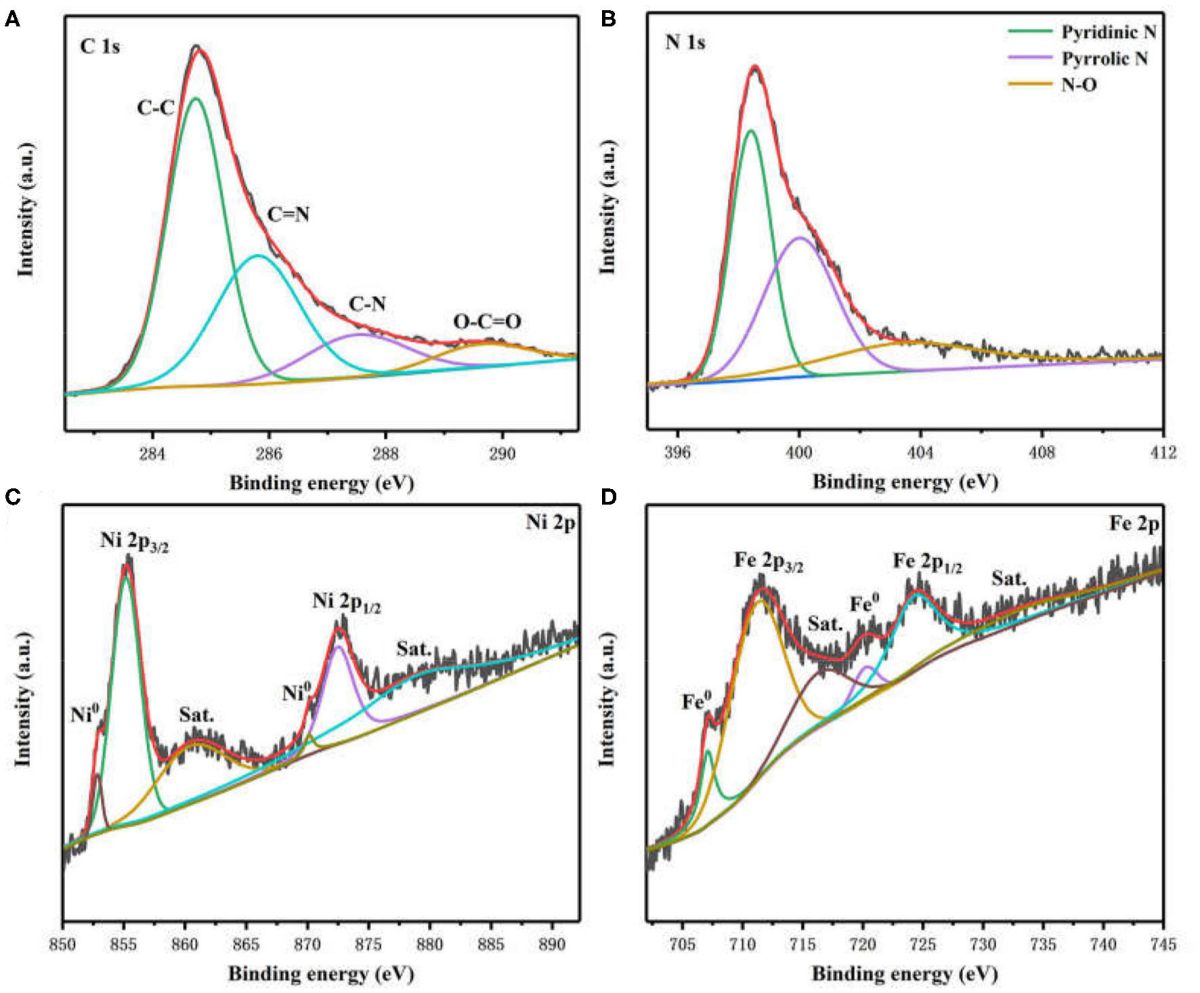
High-resolution XPS spectra of **(A)** C 1 s, **(B)** N 1 s, **(C)** Ni 2p, and **(D)** Fe 2p in Ni_3_Fe_2_@NC.

X-ray photoelectron spectroscopy (XPS) was employed to characterize the chemical states and surface structure of Ni_3_Fe_2_@NC materials. The photoelectron peaks in XPS survey spectra confirm the existence of C, N, O, Fe, and Ni species (Supplementary Figure 4). The high-resolution C 1s spectrum revealed the presence of C-C (284.7 eV), C=N (285.8 eV), C-N (287.5 eV), and O-C=O (289.6 eV) bonds in Ni_3_Fe_2_@NC (Figure 3A) (Sheng et al., 2011; Gao et al., 2014). The N 1s spectrum was deconvoluted into three peaks, which can be assigned to pyridinic N (398.5 eV), pyrrolic N (400.2 eV), and N-O (403.5 eV) (Figure 3B) (Niu et al., 2015; Han et al., 2019b). However, it is worth noting that pyridinic N can bond with metal atoms. The existence of C=N and C-N bond indicated the successful doping of N into the carbon skeleton. The two peaks at 852.8 and 855.2 eV in Ni 2p spectrum were ascribed to Ni 2p_3/2_ of Ni^0^ and Ni^2+^ (Figure 3C), respectively. The other two fitting peaks at 870.1 and 872.5 eV were attributed to Ni 2p_1/2_ of Ni^0^ and Ni^2+^ (Lee et al., 2016b; Yang et al., 2016; Huang et al., 2017; Wan et al., 2017). The two shakeup satellites of ionic state nickel located at 860.4 and 878.9 eV. The peaks in the Fe 2p spectrum included two peaks of zero-valence state (707.1 and 720.2 eV) and two peaks of high valence state (711.1 and 724.2 eV) with two peaks of shakeup satellites (716.3 and 733.2 eV), which were derived from NiFe nanoparticle and Fe-N species (Figure 3D) (Cui et al., 2013; Jiang et al., 2015; Dai et al., 2016). In general, these aforementioned results demonstrated the presence of NiFe alloy and the metal-N coordination in prepared Ni_3_Fe_2_@NC/CC electrode.

**Figure 4:**
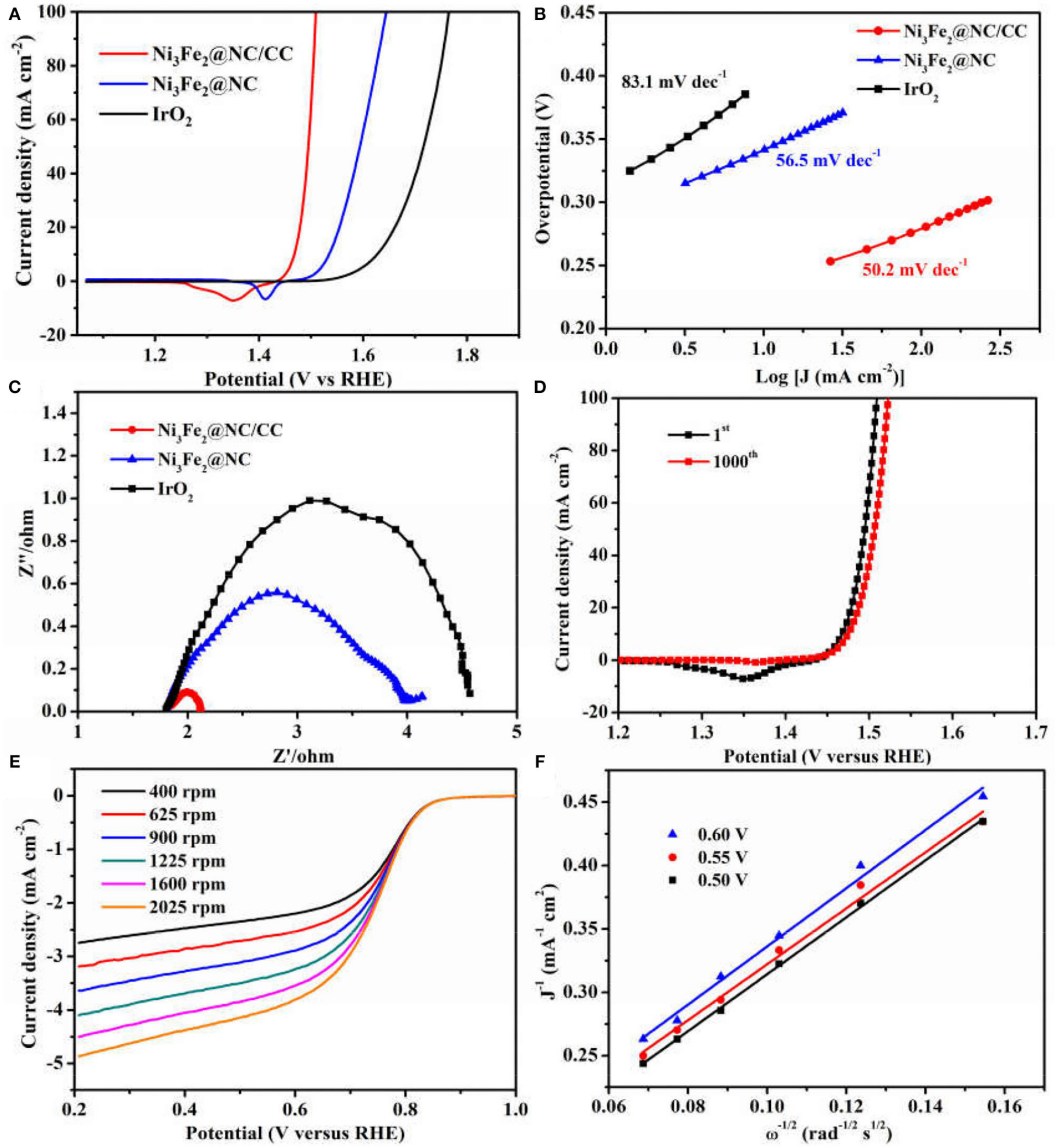
**(A)** OER polarization curves of Ni_3_Fe_2_@NC/CC, Ni_3_Fe_2_@NC, and IrO_2_. **(B)** Corresponding Tafel plots. **(C)** Corresponding EIS spectra. **(D)** OER LSV plots of Ni_3_Fe_2_@NC/CC before and after 1,000 cycles. **(E)** ORR polarization curves of Ni_3_Fe_2_@NC at different rotation rates. **(F)** The K-L plots for Ni_3_Fe_2_@NC at different potentials.

The electrocatalytic activities of Ni_3_Fe_2_@NC/CC were evaluated by comparing with noble metal IrO_2_ catalyst and Ni_3_Fe_2_@NC in a typical three-electrode configuration using 1.0 M KOH solution as the electrolyte. As displayed in the OER linear sweep voltammograms (LSV), Ni_3_Fe_2_@NC/CC possessed superior OER activity (Figure 4A). Ni_3_Fe_2_@NC/CC required an overpotential of 238 mV to reach a 10 mA cm^−2^ current density, which was much lower than those of Ni_3_Fe_2_@NC (340 mV) and IrO_2_ (400 mV). The OER activity of Ni_3_Fe_2_@NC/CC actually surpasses most previously reported electrocatalysts (Table 1). The fitted Tafel plots revealed that Ni_3_Fe_2_@NC/CC equipped a Tafel slope of 50.2 mV dec^−1^, lower than that of Ni_3_Fe_2_@NC (56.5 mV dec^−1^) and IrO_2_ (83.1 mV dec^−1^), which indicated its favorable reaction kinetic (Figure 4B). Electrochemical impedance spectroscopy (EIS) analysis suggested that the charge-transfer resistance (R_ct_) values were around 0.3, 2.2, and 3.4 Ω for Ni_3_Fe_2_@NC/CC, Ni_3_Fe_2_@NC and IrO_2_, respectively (Figure 4C), which was an indication that a rapid charge transfer rate in Ni_3_Fe_2_@NC/CC. This trend was in accordance with the polarization results and Tafel data. The above results show that, compared with the traditional drop-coated electrode, the integrated electrode gives excellent catalytic activity, which fully confirmed the structural advantages of the integrated electrode in improving the overall electrochemical activity. In addition to its high catalytic activity, the long-term stability of Ni_3_Fe_2_@NC/CC is also an important parameter for practical performance. As shown in Supplementary Figure 5, the catalyst Ni_3_Fe_2_@NC/CC exhibited remarkable OER stability at a constant current of 10 mA cm^−2^ after continuous operation for 200 min. The LSV curve of Ni_3_Fe_2_@NC/CC nearly overlapped the initial one after continuous 1,000 cyclic voltammetry (CV) cycles (Figure 4D). This result manifested that the Ni_3_Fe_2_@NC/CC hybrid can still maintain its activity after a large number of cycles and has fairly good stability. It can be seen from the Supplementary Figure 6 that the morphology of the Ni_3_Fe_2_@NC/CC has not changed significantly after the stability test, which further proves the strong interaction. Since bifunctional oxygen catalytic performance is required in rechargeable Zinc-air battery, the ORR activity of synthesized composites was assessed by coating on rotating disk electrodes (RDEs). As displayed in Figure 4E, Ni_3_Fe_2_@NC exhibited an onset potential of 0.87 V, a half-wave potential of 0.73 V, and a limiting diffusion current density of 4.5 mA cm^−2^ at a rotating speed of 1,600 round per minute (rpm), which is comparable to many reported bifunctional oxygen electrocatalysts (Supplementary Table 2). The fitted Koutechy-Levich plot of Ni_3_Fe_2_@NC disclosed an apparent 4-electron reaction pathway, which is deemed to a highly efficient mechanism dominating the ORR process (Figure 4F). The electrochemically active surface area (EASC) was measured by the double-layer capacitance (C_dl_) to be 2.5 mF cm^−2^ for Ni_3_Fe_2_@NC, indicating the benefits of NiFe nanoparticles and NC substrate in exposing more electrochemical active sites (Supplementary Figures 7A,B). Apart from the catalytic activity, Ni_3_Fe_2_@NC also showed remarkable ORR catalytic stability. After 9 h continuous chronoamperometric treatment, the active current retention of Ni_3_Fe_2_@NC was 80% (Supplementary Figure 8). The long-term stability was further demonstrated by the almost overlapping CV curves after 1,000 cycles (Supplementary Figure 8 inset). The aforementioned experimental results jointly confirm that the Ni_3_Fe_2_@NC/CC hybrid electrode has efficient bifunctional OER/ORR performance and long-term durability, indicating that it has promising application prospects in reversible oxygen electrocatalysis for rechargeable metal-air batteries and regenerative fuel cells.

**Table 1:** The OER performance of the recently reported highly active catalysts.

**Catalyst**	**Substrate**	**Electrolyte**	**Current density (mA cm^**−2**^)**	**Overpotential (mV)**	**References**
Ni_3_Fe_2_@NC/CC	Carbon cloth	1.0 M KOH	10	238	This work
NiFe LDH	Ni foam	1.0 M KOH	10	490	Chen et al., 2019
NiCo_2_O_4_	Ni foam	1.0 M KOH	10	270	Fang et al., 2018
Fe-doped NiO_x_	GCE	1.0 M KOH	10	310	Wu et al., 2017
NiFe_2_O_4_	GCE	0.1 M KOH	10	440	Li et al., 2015
NiCo_2_S_4_ NW	Ni foam	1.0 M KOH	10	400	Sivanantham et al., 2016
Ni_3_FeN-NPs	GCE	1.0 M KOH	10	280	Jia et al., 2016
Ni-Co-Fe NNCs	Copper	1.0 M KOH	10	316	Barati Darband et al., 2019
a-Co_2_Fe	GCE	1.0 M KOH	10	290	Zhu et al., 2020
Co_3_FeS_1.5_(OH)_6_	GCE	0.1 M KOH	10	358	Wang et al., 2017
NiCoP/C nanoboxes	GCE	1.0 M KOH	10	330	He et al., 2017

**Figure 5:**
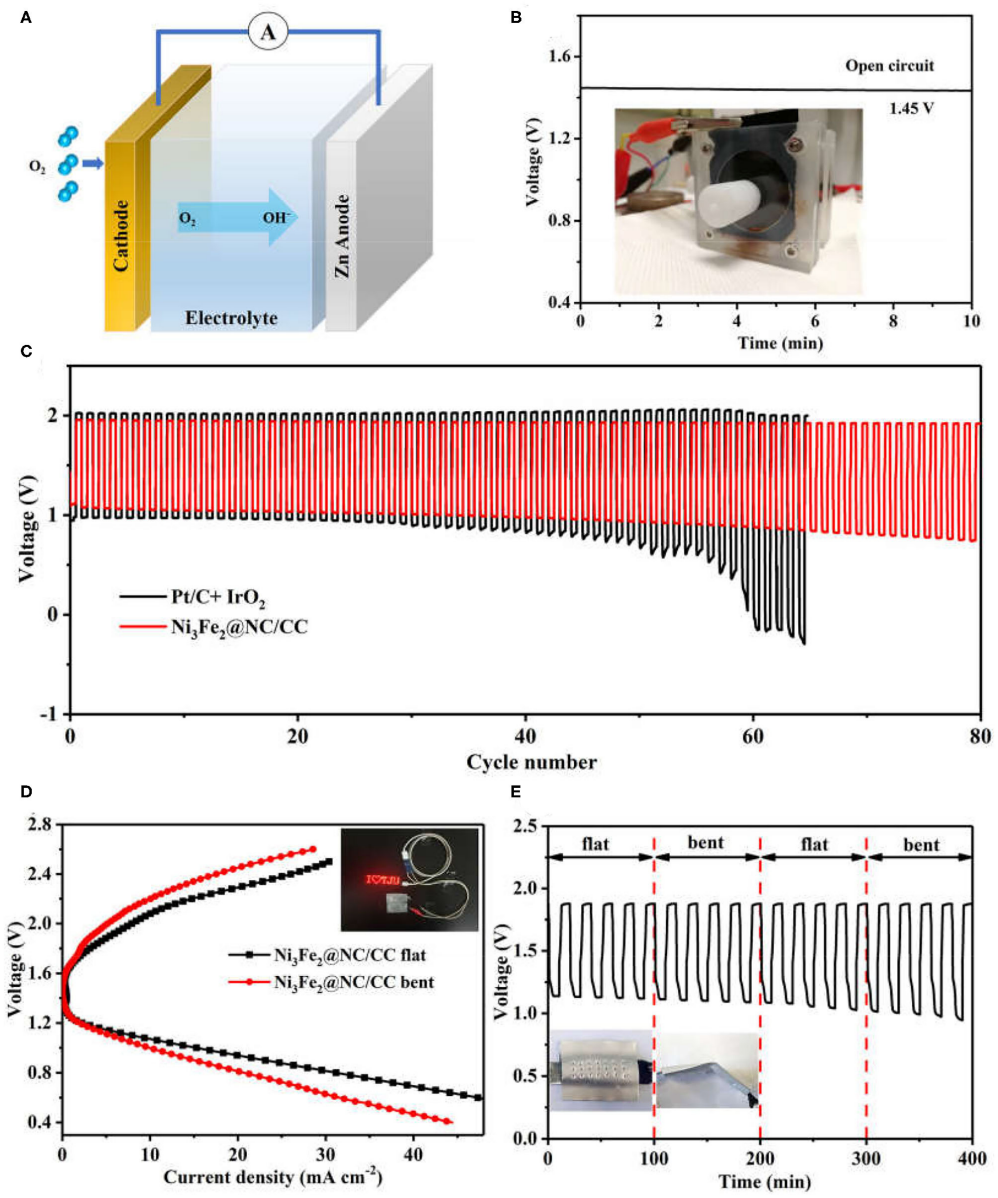
**(A)** Schematic configuration of an aqueous Zinc-air battery. **(B)** Open-circuit voltages of Ni_3_Fe_2_@NC/CC based zinc-air batteries. Inset shows a Zinc-air battery under test. **(C)** Cycling performance of rechargeable zinc-air batteries. **(D)** Discharge and charge polarization curves and **(E)** cycling performance of Ni_3_Fe_2_@NC/CC-based flexible battery at 2 mA cm^−2^. Inset shows a LED screen powered by Ni_3_Fe_2_@NC/CC-based zinc-air batteries.

A home-made liquid zinc-air battery was constructed to evaluate the practical electrochemical performance of Ni_3_Fe_2_@NC/CC, in which a zinc plate was served as the anode, the Ni_3_Fe_2_@NC/CC integrated electrode as the air cathode, and 6.0 M KOH and 0.2 M ZnCl_2_ as the electrolyte (Figure 5A). The alkaline zinc-air batteries based on Ni_3_Fe_2_@NC/CC materials exhibit a steady open-circuit voltage of 1.45 V (Figure 5B). In addition, with the current density of 5 mA cm^−2^ and the duration of each cycle of 20 min, the cycle chargeability of the secondary battery was further studied (Figure 5C). It is worth noting that under the catalysis of Ni_3_Fe_2_@NC/CC electrode, the discharge voltage of the battery is 1.11 V, the charging voltage is 1.95 V, the voltage gap is 0.84 V, and the energy efficiency is 56.9%. Small voltage decay is observed after 80 cycles for Ni_3_Fe_2_@NC/CC cathode, which reflects the excellent rechargeability and is much better than the precious metal Pt/C-IrO_2_ catalyst (<50 cycles). The discharge specific capacity of a primary Zinc-air battery with Ni_3_Fe_2_@NC/CC cathode is 655 mA h g^−1^ at 20 mA cm^−2^ based on the mass of consumed zinc (Supplementary Figure 9). Encouraged by the potential application for portable and wearable devices, a flexible solid-state zinc-air battery was assembled with zinc plate anode, PVA-KOH electrolyte and Ni_3_Fe_2_@NC/CC integrated cathode. The open circuit voltage of solid zinc-air battery promoted by Ni_3_Fe_2_@NC/CC can reach 1.37 V, again indicating the efficient ORR activity of the integrated cathode (Supplementary Figure 10). As shown in Figure 5D inset, a light emitting diode (LED) screen was powered by two Ni_3_Fe_2_@NC/CC-based solid flexible Zinc-air batteries in series. The voltage-current polarization curves revealed that the Ni_3_Fe_2_@NC/CC cathode possessed a good charge-discharge performance (Figure 5D). The discharge voltage at 10 mA cm^−2^ is 1.09 V and the charge voltage is 2.08 V when the battery is flat. Moreover, the battery exhibits considerable flexibility. When the assembled battery is bent about 30°, the polarization curve shows that the discharge voltage is 1.07 V at a current density of 10 mA cm^−2^, which is only 1.8% lower than the discharge voltage in the flat state of the battery, and the charging voltage is 2.20 V, which is 5.7% higher. As shown in Figure 5E, the assembled battery can be alternately flat and bent (once every 5 charge-discharge cycles). Under a large mechanical strain, the battery can maintain a good charge and discharge cycle under continuous charging and discharging conditions. These results fully prove the good flexibility and cycle stability of Ni_3_Fe_2_@NC/CC-based rechargeable zinc-air batteries.

## Conclusion

In conclusion, Ni_3_Fe_2_@NC/CC integrated electrode was prepared by uniformly loading nickel-iron bimetallic nanoparticles on nitrogen-doped carbon nanostructures grown on carbon cloth support by chemical precipitation and then high-temperature calcination. The preparation technology of the integrated electrode with the catalyst uniformly supported on the conductive carrier is simple, and the electrode presents high catalytic activity and good stability. The prepared Ni_3_Fe_2_@NC/CC shows enhanced OER/ORR activity and durability either in electrochemical half-reaction tests or reversible zinc-air batteries, which is superior to noble metals and many previously developed electrocatalysts. Further experimental analysis demonstrates the advantages of *in-situ* integrated electrode in facilitating the electrode transfer and strong interaction between the active materials and the current collector, thereby promoting the electrocatalytic performance. Our findings provide a simple strategy for the preparation of bimetallic nanocomposites and open up a new way for the development of promising hybrid catalysts for electrochemical and energy-related applications.

## Data Availability Statement

The original contributions presented in the study are included in the article/Supplementary Material, further inquiries can be directed to the corresponding author/s.

## Author Contributions

HH and XL conducted the experiments and write the manuscript. CT helped with operating the experiments and data analysis. JL and XH interpreted the results. XH and WH supervised the research. All authors approved the submission of final manuscript.

## Supporting Information

SEM images and XRD pattern of precursors, XRD pattern, EDS, survey XPS, CVs, C_dl_ and chronoamperometric curves of Ni_3_Fe_2_@NC/CC. Primary discharge curve of zinc-air battery, Table of comparison of OER/ORR electrocatalytic performance.

## Conflict of Interest

The authors declare that the research was conducted in the absence of any commercial or financial relationships that could be construed as a potential conflict of interest.
