# Supplementary material for: Preparation of Ni3Fe2@NC/CC Integrated Electrode and Its Application in Zinc-Air Battery
Source: Front Chem. 2020 Nov 9;8:575288. doi: 10.3389/fchem.2020.575288 (PMC7693560; doi:10.3389/fchem.2020.575288)
Supplement: Supplementary file 1 [file Data_Sheet_1.doc]

**Supporting Information**

Preparation of Ni_3_Fe_2_@NC/CC Integrated Electrode and Its Application in Zinc-air Battery

*Hui Hu,^1,2^ Xiaofei Ling,^2^ Chaogui Tan,^1^ Jianguo Lin,^1^ Xiaopeng Han,^2,*^ Wenbin Hu,^2,*^*

^1^School of Materials Science and Engineering, Xiangtan University 411105, China; ^2^Tianjin Key Laboratory of Composite and Functional Materials, School of Materials Science and Engineering, Tianjin University, Tianjin 300072, China

**
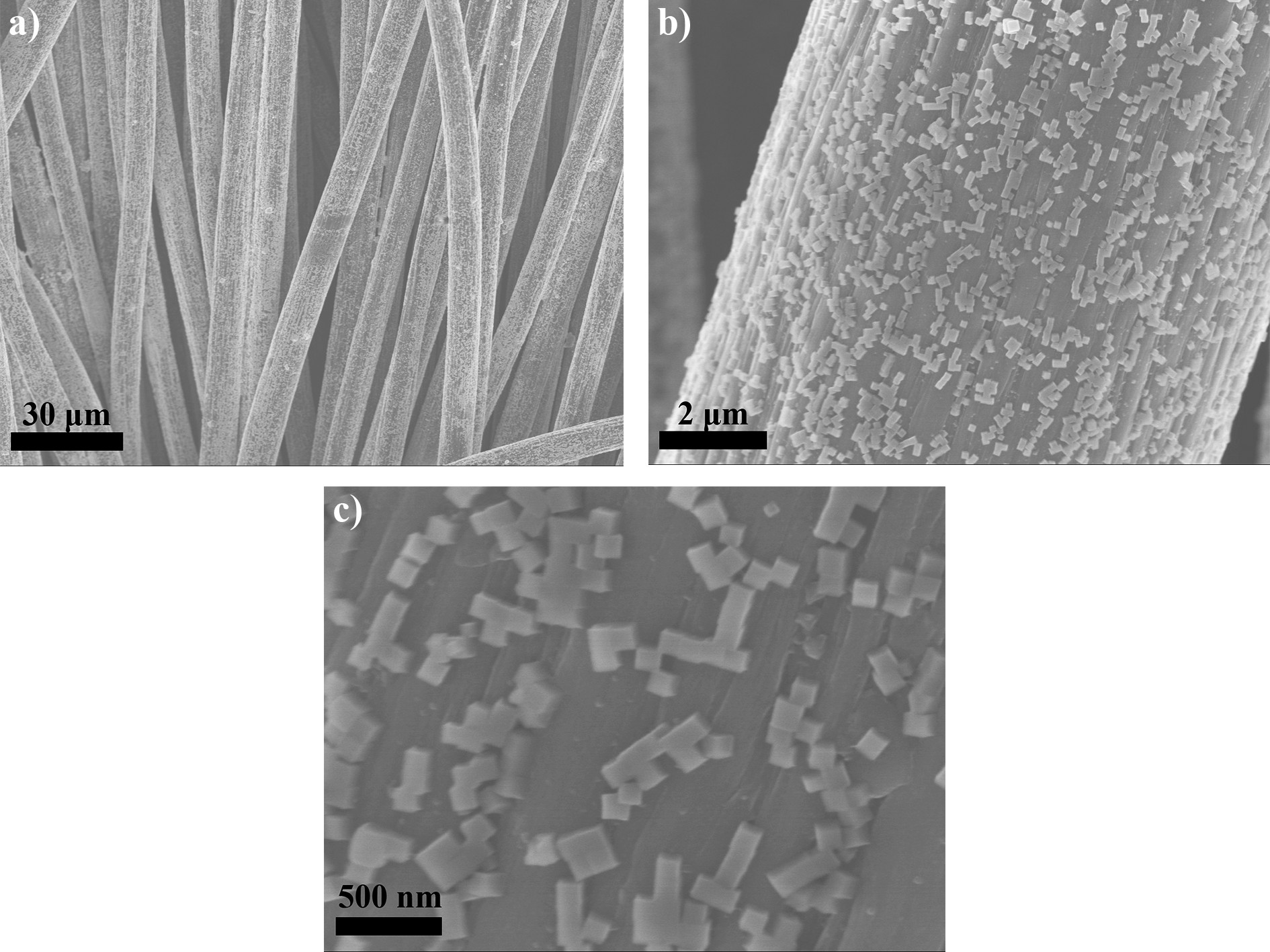
**

**Figure S1.** a-c) SEM images from low to high magnification of the precursors.

**
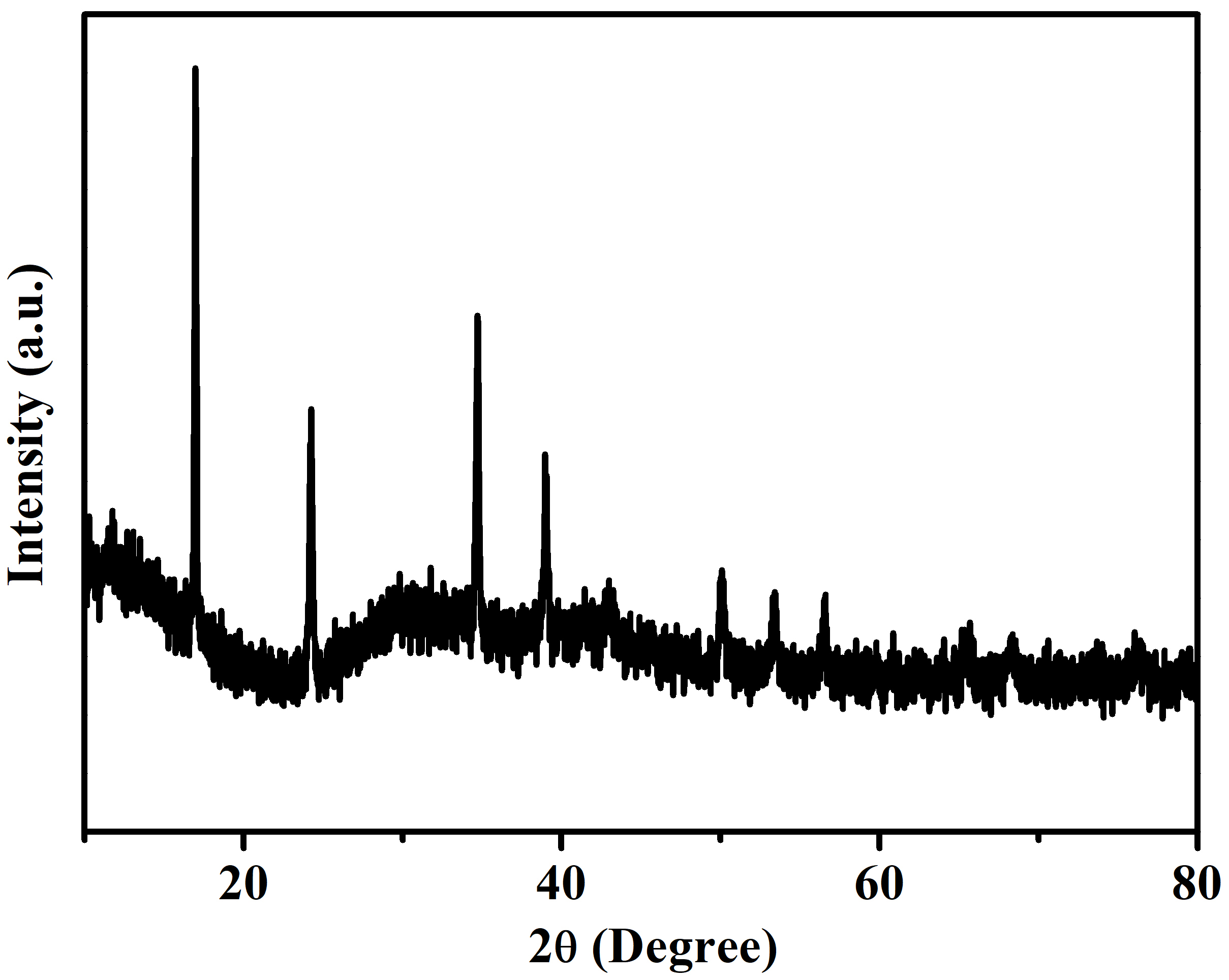
**

**Figure S2.** XRD pattern of the precursors.


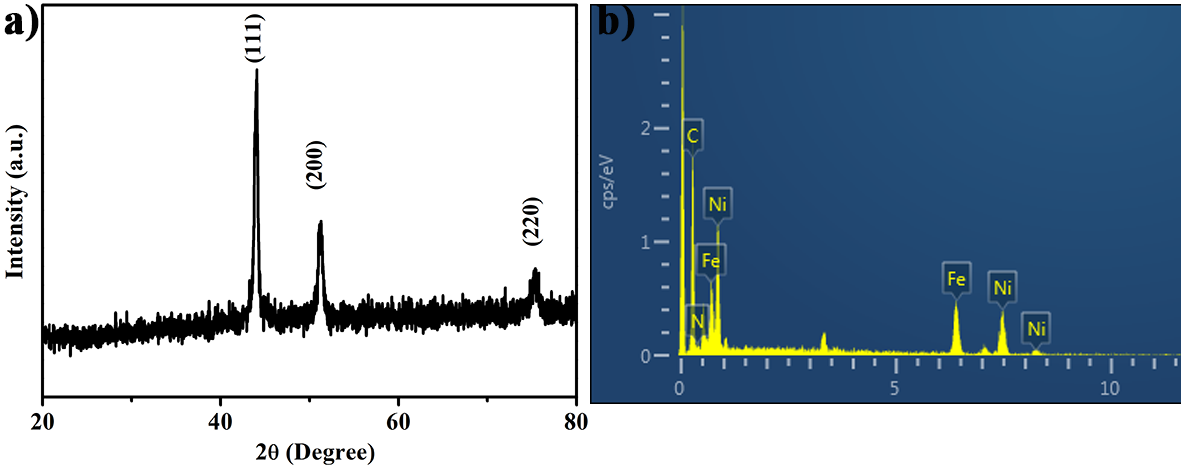


**Figure S3.** a) XRD pattern and b) EDS of Ni_3_Fe_2_@NC.


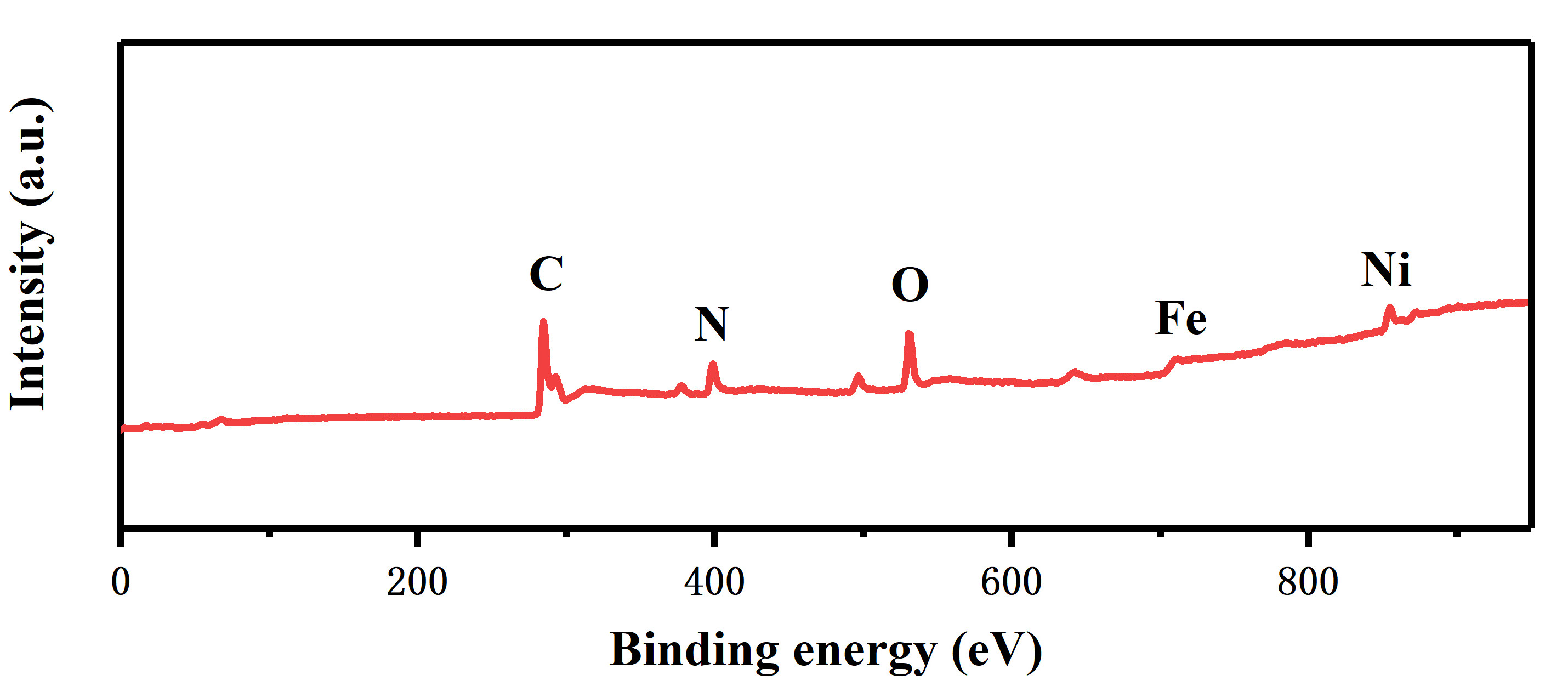


**Figure S4.** Survey XPS of Ni_3_Fe_2_@NC.


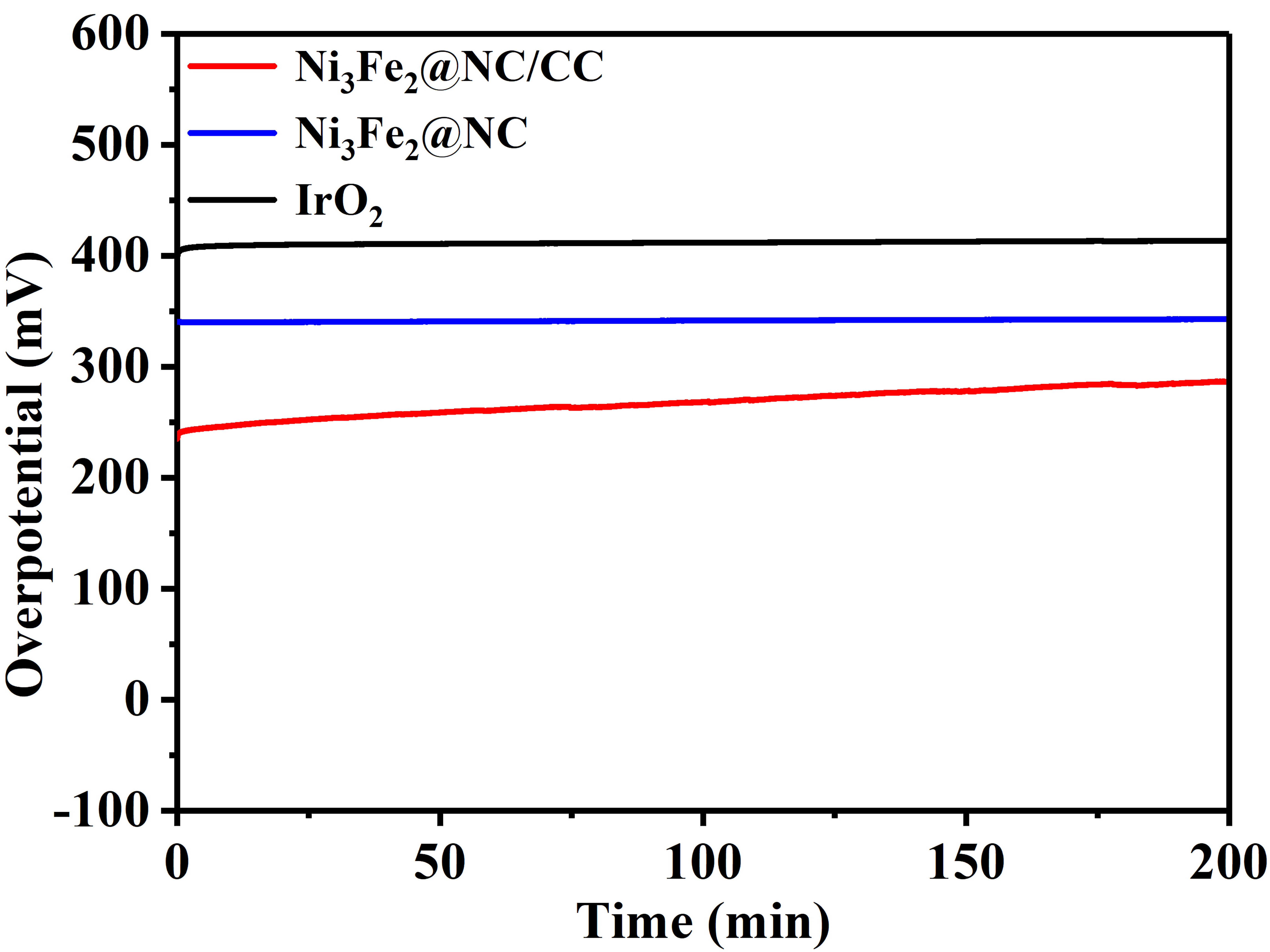


**Figure S5.** Chronopotentiometry response of Ni_3_Fe_2_@NC/CC, Ni_3_Fe_2_@NC, and IrO_2_ at a 10 mA cm^−2^.


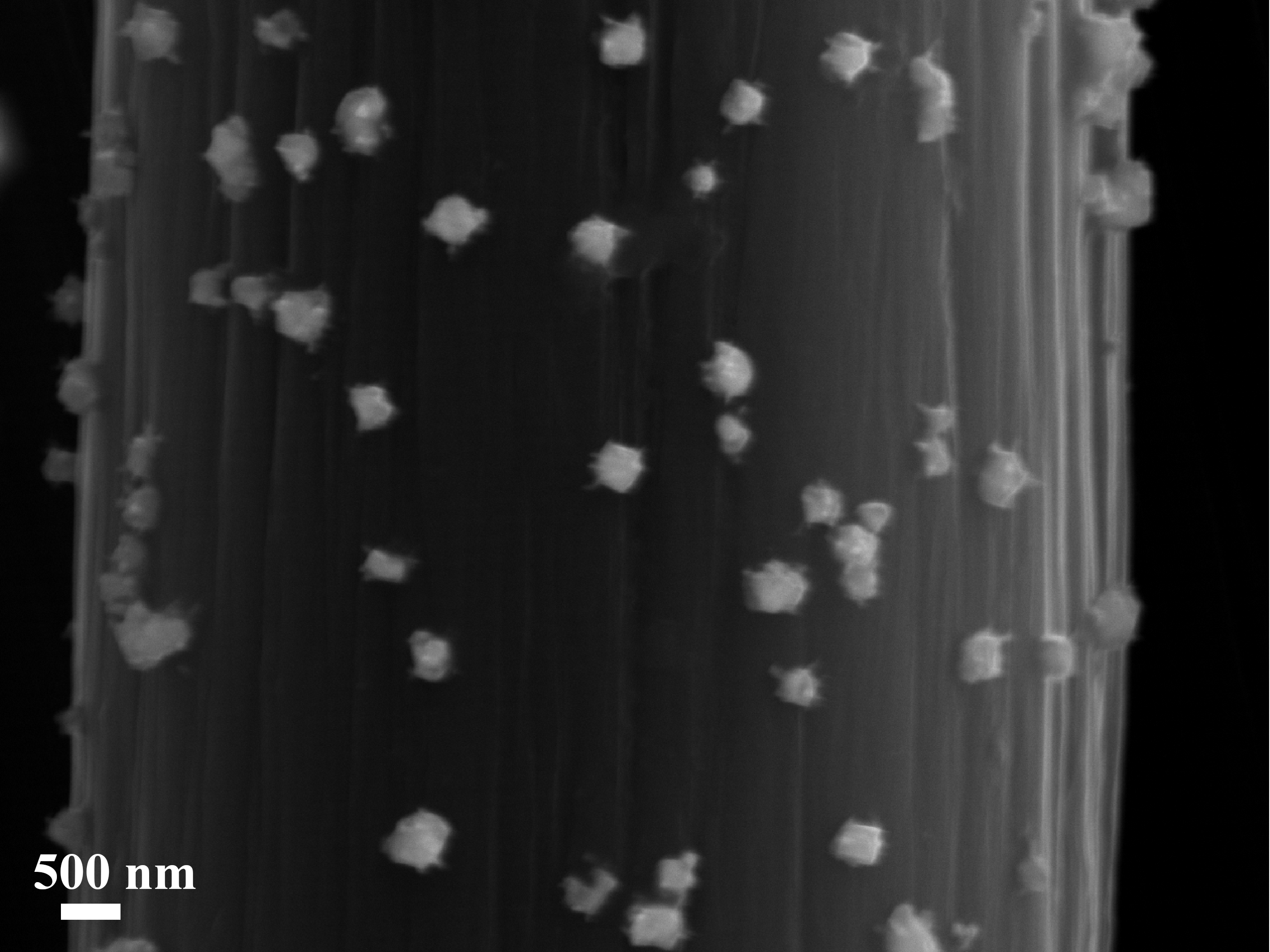


**Figure S6.** SEM image of the Ni_3_Fe_2_@NC/CC integrated electrode after the OER stability test.


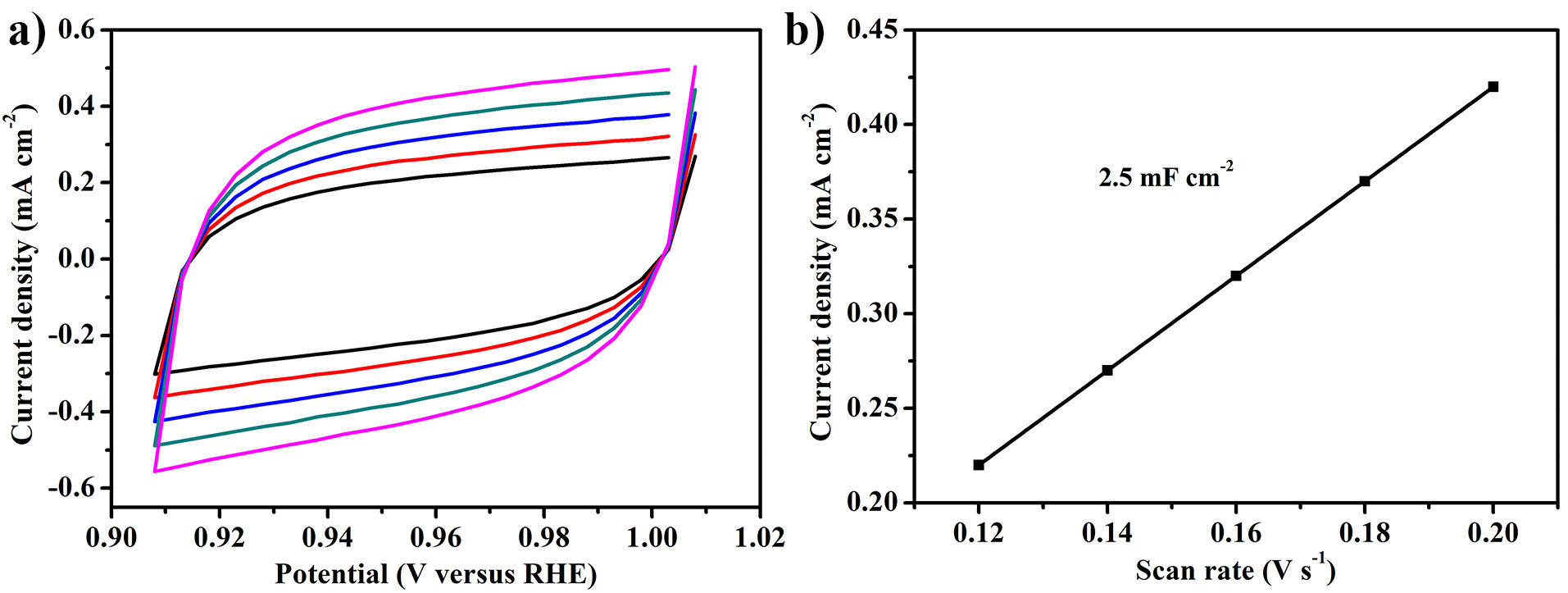


**Figure S7.** a) Cycle voltammograms from 0.91 to 1.01 V versus RHE for Ni_3_Fe_2_@NC in 0.1 M KOH at scan rates of 0.12, 0.14, 0.16, 0.18, and 0.20 V s^-1^, respectively. b) Dependence of current densities as a function of scan rates for Ni_3_Fe_2_@NC.


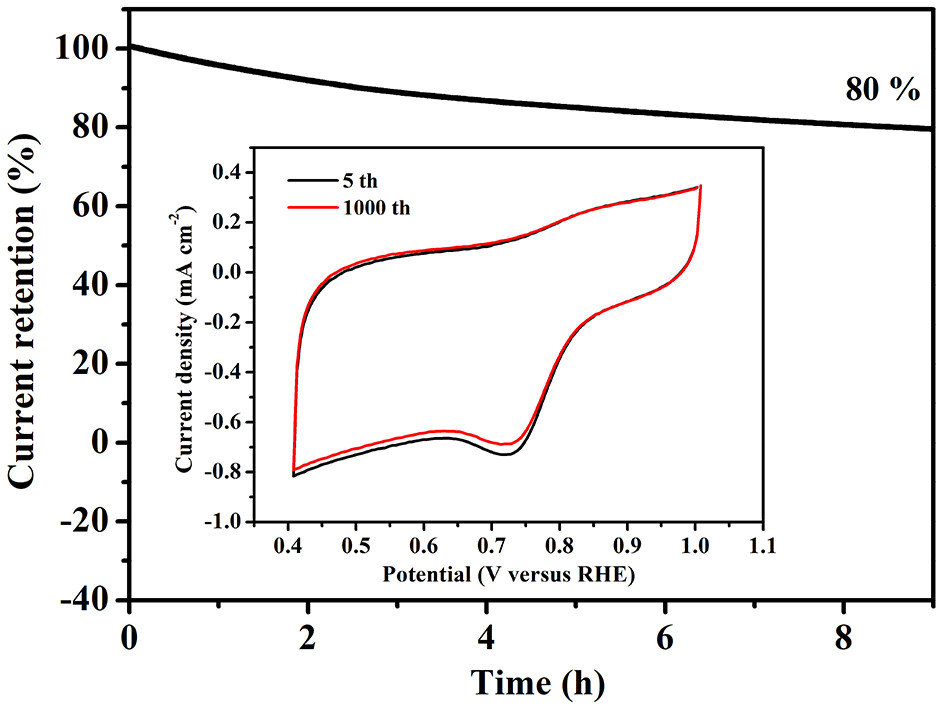


**Figure S8.** Chronoamperometric curves of Ni_3_Fe_2_@NC at 0.6 V vs. RHE. Inset shows the prolonged CVs of Ni_3_Fe_2_@NC at 400 rpm at a scan rate of 250 mV s^-1^.


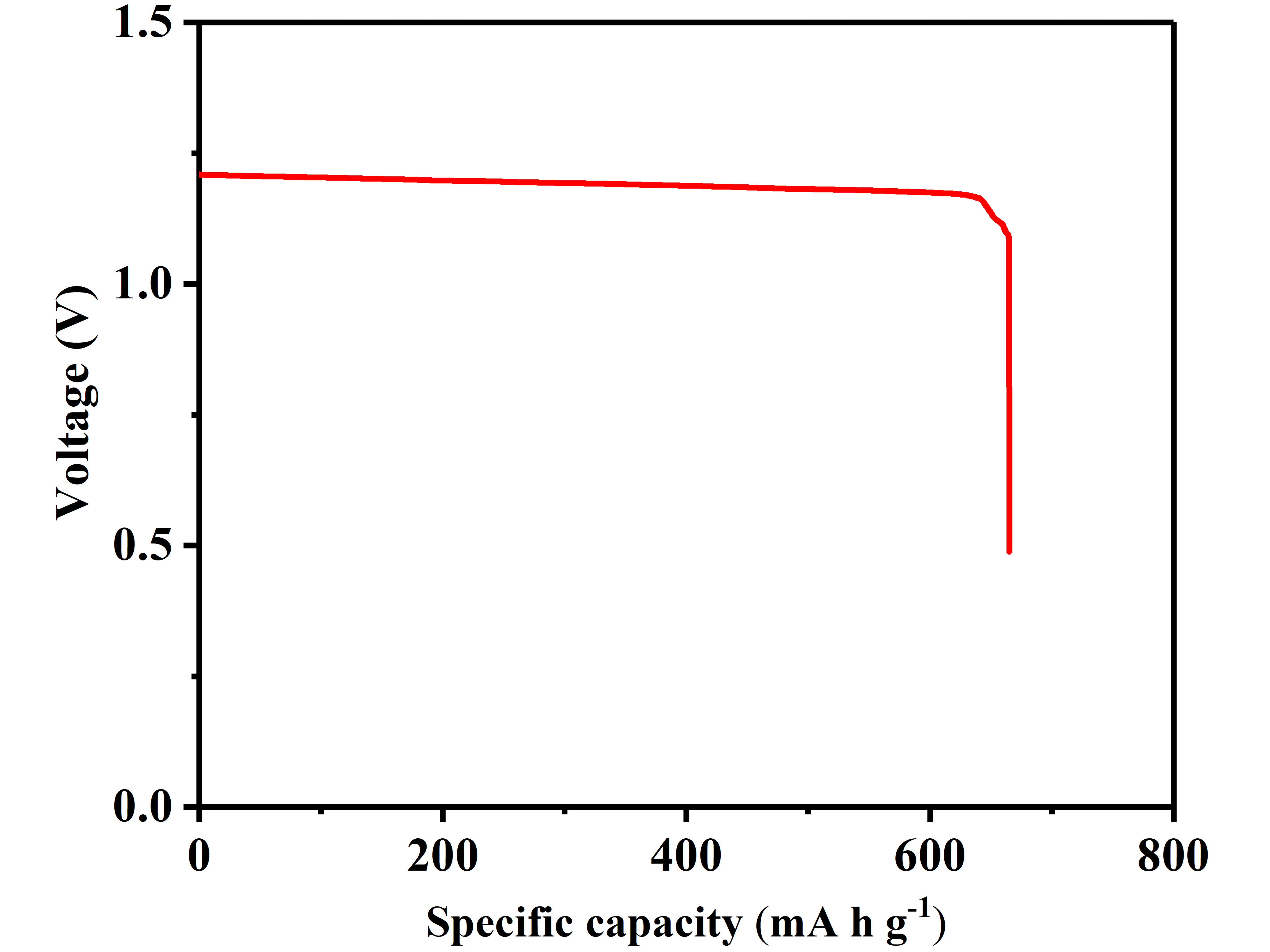


**Figure S9.** The specific capacity of zinc-air battery catalyzed by Ni_3_Fe_2_@NC/CC at 20 mA cm^-2^ based on the weight of consumed Zn.


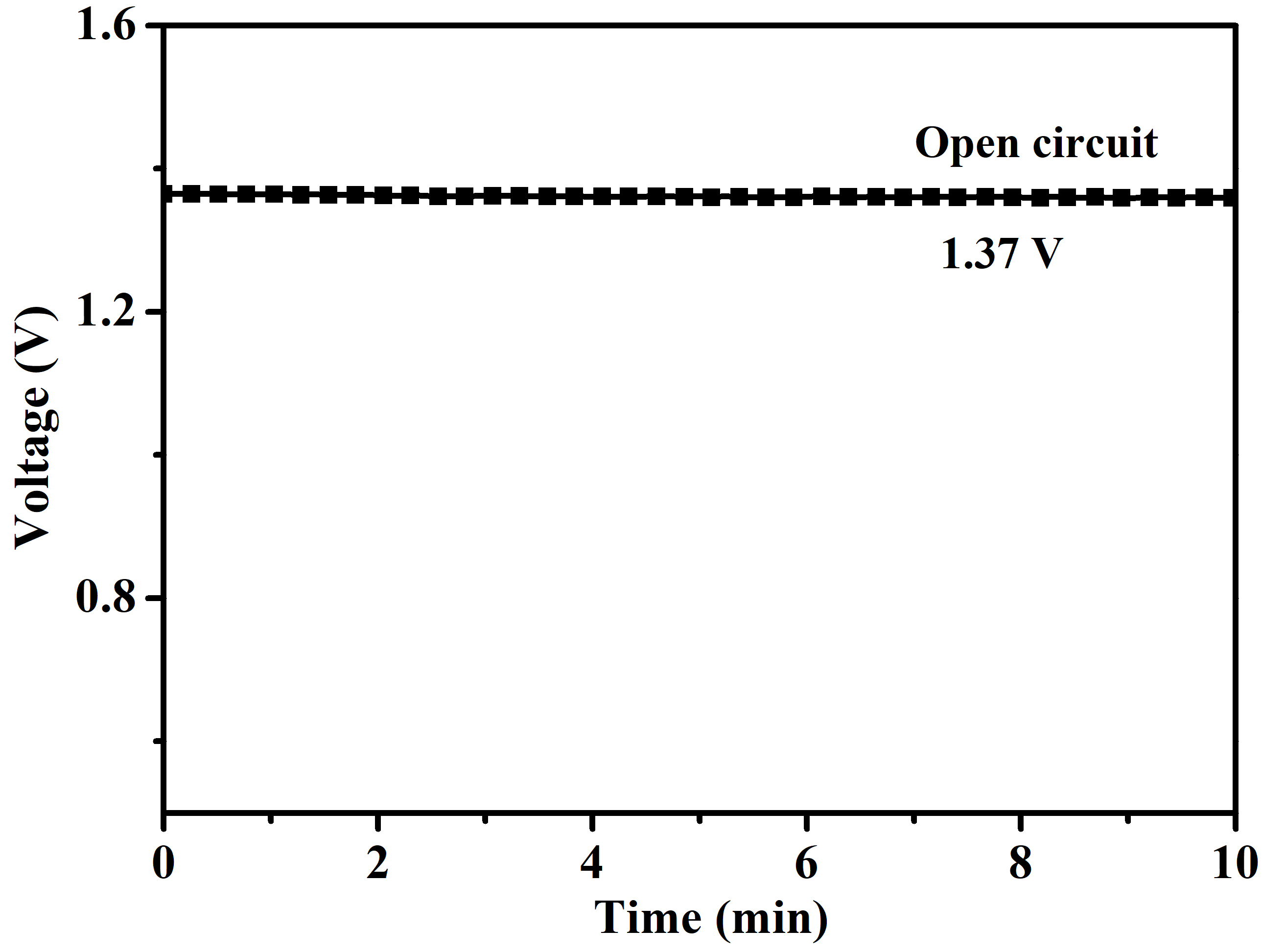


**Figure S10.** OCP plot of the solid flexible zinc-air battery with Ni_3_Fe_2_@NC/CC cathode.

**Table S1.** The content of Ni and Fe in Ni_3_Fe_2_@NC obtained by EDS.

| **Element** | **wt%** |
| --- | --- |
| Ni | 30.03 |
| Fe | 20.48 |

**Table S2** Comparison of OER/ORR electrocatalysis performance for Ni_3_Fe_2_@NC/CC in this work with recently reported bifunctional catalysts.

| **Catalyst** | **E_j=10_**  **(V vs RHE)** | **E_1/2_**  **(V vs RHE)** | **ΔE**  **(V vs RHE)** | **Refs** |
| --- | --- | --- | --- | --- |
| Ni_3_Fe_2_@NC/CC | 1.468 | 0.73 | 0.738 | This work |
| CoNi-NPs/NC | 1.67 | 0.64 | 1.03 | Adv. Mater. 2019, 31, e1905622 |
| NiCo_2_O_4_ | 1.64 | 0.72 | 0.92 | Nanoscale. 2014, 6, 3173-3181 |
| Ni_x_O_y_/NC | 1.64 | 0.71 | 0.93 | Angew. Chem. Int. Ed. 2014, 53, 8508-8512 |
| Co@Co_3_O_4_/NC-2 | 1.64 | 0.74 | 0.90 | Angew. Chem. Int. Ed. 2016, 55, 4087-4091 |
| P-doped g-C_3_N_4_ /CFP | 1.63 | 0.67 | 0.96 | Angew. Chem. Int. Ed. 2015, 54, 4646-4650 |
| Fe@N-C900 | 1.76 | 0.76 | 1.00 | Nano. Energy. 2015, 13, 387-396 |
| CoFe_2_O_4_/C | 1.72 | 0.71 | 1.01 | Appl. Surf. Sci. 2017, 403, 51-56 |
| Co-N/C 800 | 1.74 | 0.78 | 0.96 | J. Mater. Chem. A. 2016, 4, 16920-16927 |
